# Expedient syntheses of the N-heterocyclic carbene precursor imidazolium salts IPr·HCl, IMes·HCl and IXy·HCl

**DOI:** 10.1186/1860-5397-3-22

**Published:** 2007-08-28

**Authors:** Lukas Hintermann

**Affiliations:** 1 Institute of Organic Chemistry, RWTH Aachen University, Landoltweg 1, D-52074 Aachen, Germany

## Abstract

The 1,3-diaryl-imidazolium chlorides IPr·HCl (aryl = 2,6-diisopropylphenyl), IMes·HCl (aryl = 2,4,6-trimethylphenyl) and IXy·HCl (aryl = 2,6-dimethylphenyl), precursors to widely used N-heterocyclic carbene (NHC) ligands and catalysts, were prepared in high yields (81%, 69% and 89%, respectively) by the reaction of 1,4-diaryl-1, 4-diazabutadienes, paraformaldehyde and chlorotrimethylsilane in dilute ethyl acetate solution. A reaction mechanism involving a 1,5-dipolar electrocyclization is proposed.

## Background

**Figure 1:**
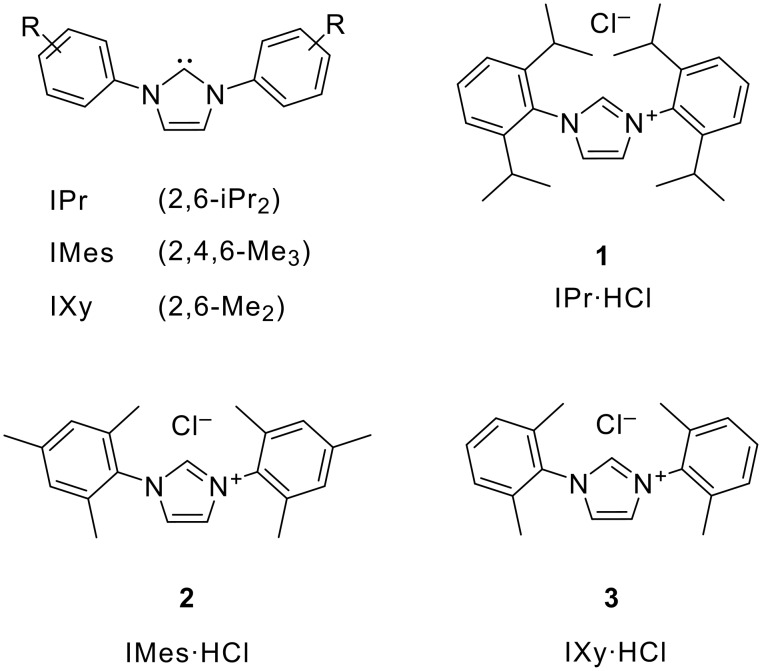
The carbenes IPr, IMes, IXy and their imidazolium salt precursors

Imidazolylidene carbenes have been investigated as ligands in coordination chemistry, as powerful steering/controlling elements in transition-metal catalysis,[1–2] and more recently as metal-free catalysts for organic reactions[3–4]. Some prominent members of the family of N-heterocyclic carbenes (NHC) are the sterically encumbered imidazolylidenes IPr and IMes (Figure 1), which can also be considered as analogues of bulky and electron-rich tertiary phosphanes. In contrast to the latter, their synthesis does not involve air-sensitive or pyrophoric organometallic reagents, and they are conveniently stored and used in the form of their air-stable precursors, namely the imidazolium salts IPr·HCl (1,3-bis-{2,6-diisopropylphenyl}imidazolium chloride; **1**) or IMes·HCl (1,3-bis-{2,4,6-trimethylphenyl}imidazolium chloride; **2**) (Figure 1).

At present, there is a slightly ironic situation: even though salts **1** and **2** are in high demand and used by many researchers, information about their synthesis is scattered over the literature, and standard references for satisfactory, high-yielding (e.g., >60%) preparations are not available. The present work introduces a simple, reliable and economic synthesis of IPr·HCl (**1**), IMes·HCl (**2**) and IXy·HCl (**3**). The procedure should be especially valuable to those who want to prepare these useful salts on large scale, rather than obtaining them from commercial suppliers.

**Scheme 1:**
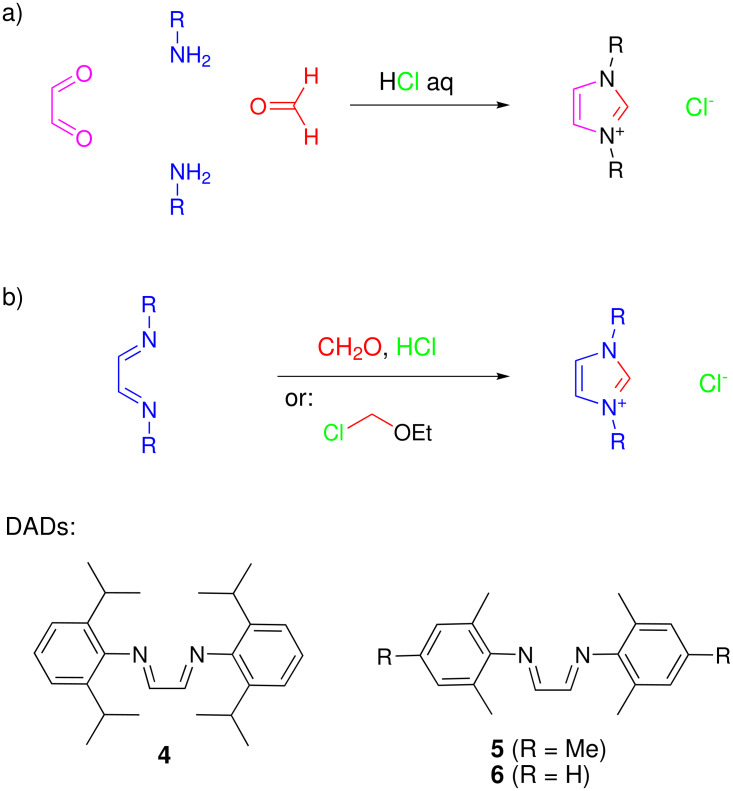
Synthetic routes to and diazadiene precursors for imidazolium salts.

**Scheme 2:**
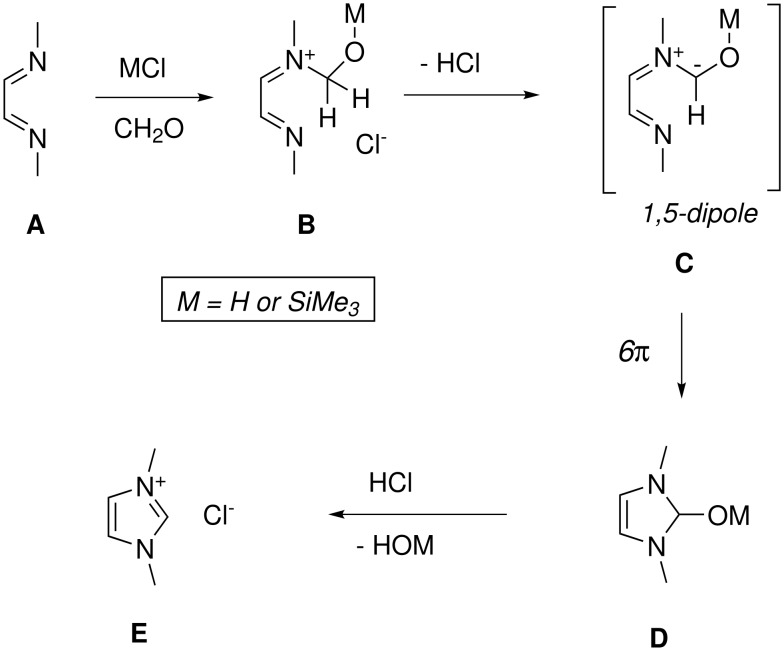
The imidazolium salt synthesis as a 1, 5-dipolar electrocyclization.

A synthesis of *N*,*N*-disubstituted symmetric imidazolium salts by condensation of glyoxal, two equivalents of aliphatic or aromatic amine, and an equivalent of paraformaldehyde in the presence of hydrochloric acid was reported by Arduengo in a patent in 1991 (Scheme 1a). [5] It is successful for a range of alkyl- and arylamines, but the original document gave only a few examples and limited details on product purification. In fact, one characteristic of the protocol is the generation of dark-brown impurities which render product purification tedious. Thus, the synthesis of IMes·HCl (**2**) according to the Arduengo protocol is followed by extensive solvent washes, resulting in a low yield (40%). [6–7] Both the Arduengo and Nolan groups noted that this synthetic protocol cannot be extended to the sterically more hindered IPr·HCl (**1**). [8–9] Instead, Arduengo and coworkers found that the reaction of the 1,4-diaryl-1,4-diazadienes (DADs, or glyoxal imines) **4** and **5** with chloromethyl-ethylether (THF, 40°C/16 h or 23°C/5 d) gave pure IPr·HCl (**1**) or IMes·HCl (**2**) in 47% or 40% yield, respectively (Scheme 2b). [8]

In parallel, Nolan and coworkers obtained IPr·HCl (**1**) from DAD **4** and paraformaldehyde in toluene, using HCl/dioxane (4 M) as source of the counter-ion under homogeneous conditions (36 h, r.t., 47% yield) [9–10]. A corresponding procedure for **2** has not been reported. Noels and coworkers have prepared IXy·HCl (**3**) from DAD **6**, paraformaldehyde and HCl/dioxane in THF (r.t., 4 h, 55%), and similarly 1,3-di-*ortho*-tolyl-imidazolium chloride (32% yield) and 1,3-bis-*para*-biphenyl-imidazolium chloride (72% yield). [11] Recently, a patent by Nolan discloses a synthetic procedure starting from DADs **4**/**5** (1 equiv), paraformaldehyde (1.3 equiv) and HCl/dioxane (1.6 equiv) in ethyl acetate. Following a neutralization and a purification step, **1** or **2** were obtained in 70% or 66% yield, respectively. [12]. Judging from various literature references, it appears that IPr·HCl (**1**) is now usually prepared by Nolan's first route from **4**,[10] and IMes·HCl (**2**) by Arduengo's one-pot [5–6] or the chloromethylether route. [8]. In any case, it is difficult to find optimal synthetic procedures for these important compounds, and the most popular syntheses are reported to give yields in the 40% range.

## Results

Literature precedence implies that the two step synthesis via diazadienes (Scheme 2b) is advantageous for the final purity of the imidazolium products. We have obtained the glyoxal imines **4**–**6** following established procedures.[8,10,13–15] Our experiments on the synthesis of **1** started with the Nolan procedure (condensation of **4** and paraformaldehyde in toluene), but we whished to replace the reagent HCl/dioxane (4 M) by a more readily available and cheaper source of the chloride counter ion. The choice fell on chlorotrimethylsilane (TMSCl), the "silyl version" of HCl, which is easily measured and handled. The current pricing of TMSCl is 8 €/mol, as opposed to 80–100 €/mol for HCl/dioxane (4 M). The reaction optimization went as follows: Initial experiments used two equivalents of TMSCl in hot toluene, assuming the validity of equation 1 for the reaction:

[1]
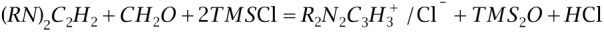


This protocol gave IPr·HCl (**1**) in satisfactory yield as almost colorless microcristalline powder (Table 1, entry 1), and also worked well for the synthesis of IMes·HCl (**2**; entry 7). However, it gave mixed results when applied to IXy·HCl (**3**), with reaction solutions turning deep brown, and the solid precipitate being accompanied by impurities. We suspected that HCl as a strongly acidic byproduct (see equation 1) was responsible for side reactions. The amount of TMSCl was therefore reduced to one equivalent, which assures neutral reaction conditions, but also means that water is liberated in the course of the reaction (equation 2):

**Table 1:** Optimization of the TMSCl-induced imidazolium salt synthesis

entry	starting material	stoichiometry^a^	scale [mmol]	solvent [mL/mmol]	T [°C]	time [h]	product	yield [%]

1	**4**	1:1:2^b^	6.6	PhMe (7.5)	80+r.t.	4+10	**1**	61
2	**4**	1:1:1.9	26.6	PhMe (5.6)	75–80	3	**1**	67
3	**4**	1:1:1.9	122	PhMe (2.9)	70	5	**1**	<40^c^
4	**4**	1:1:1.05	135.5	PhMe (7.4)	85	2.5	**1**	65
5	**4**	1:1:1.04	5.3	THF (2.8)	70	4	**1**	31^d^
6	**4**	1:1:1	134	EtOAc (9.5)	70	2.75	**1**	81
7	**5**	1:1:2	7.5	PhMe (4)	75–80	3	**2**	64
8	**5**	1:1:1	7.5	EtOAc (9)	70	1.5	**2**	69
9	**6**	1:1:2	25	PhMe (6)	80	3.5	**3**	43^e^
10	**6**	1:1.05:1.35	10	PhMe (2)	80	2.25	**3**	-^f^
11	**6**	1.04:1:1.09	59	PhMe (7)	85	1	**3**	-^f^
12	**6**	1:1:1.2	10	THF (2)	r.t.	24	**3**	53
13	**6**	1:1:1	19	PhMe/THF (8.4)	85	3.5	**3**	88
14	**6**	1:1:1	19	EtOAc (8.4)	80	1.5	**3**	89.5

a) Diazadiene/paraformaldehyde/TMSCl. b) Addition of TMSCl in two portions, in all other cases slow, dropwise addition. c) Impure product. d) Yield after recrystallization from acetone/tBuOMe; raw yield 60%. e) Losses due to washing away of colored impurities with acetone. f) Brown, impure material was obtained.

[2]
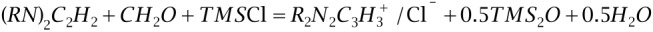


DAD **4** was still cyclized to **1** (entry 4), but in reactions of **6**, the water of condensation separated as drops which accumulated both acid (from TMSCl + H_2_O) and reaction intermediates, producing brown resins. We reasoned that the water of condensation had to be removed either chemically or simply by homogeneous dissolution in the reaction mixture. Indeed, by adding 30% of tetrahydrofurane (THF) to the reaction solvent (entry 13), IXy·HCl (**3**) was precipitated in very high yield and satisfactory purity. Since THF is relatively expensive, we hoped to find another one-component reaction solvent instead, whose polarity would be low enough to precipitate the imidazolium salts in pure form, but also sufficiently high in order to dissolve the liberated water of condensation. Ethyl acetate fulfilled these requirements and gave optimal results in all imidazolium salt syntheses (entries 6,8,14). In the course of scale-up, the reactant concentration was identified as another important parameter (entry 2 vs 3; entries 5,10). The final, recommended procedure for the synthesis of 1,3-diarylimidazolium chlorides consists in combining the reactants in stoichiometric quantities in ethyl acetate (7–10 mL/mmol) at 70°C. This simple protocol gave IPr·HCl (**1**), IMes·HCl (**2**) and IXy·HCl (**3**) in pure form and high yields (Table 1, entries 6,8,14). See Supporting Information File 1 for full experimental data.

## Discussion

The synthesis of N,N-diarylimidazolium salts according to the Arduengo three component condensation [5] produces strongly colored reaction mixtures, from which the products are obtained as impure raw materials. [5–8,11] The biphasic aqueous/organic reaction conditions of that protocol have been identified as problematic, and more recent syntheses start from preformed 1,4-diazabutadienes, and either paraformaldehyde/HCl/dioxane [9–10] or chloromethyl ethers [8] or other chloromethyl-derivatives (*e.g.*, ClCH_2_OPiv) in combination with silver salts of non-coordinating counter ions. [16–17] We have now found that the use of TMSCl as chloride donor instead of HCl, ethyl acetate as the solvent, and an appropriate dilution of the reactants lead to a reliable synthetic procedure for the imidazolium salts **1–3**. However, in terms of generality, syntheses of imidazolium salts remain capricious in nature, as our conditions were not successful for other DADs such as those derived from *ortho*-toluidine, *para*-toluidine or *para*-chloroaniline. These gave strongly colored reaction mixtures, from which dark solids separated, which we have not attempted to purify. What is the mechanistic basis behind such differences of the reaction outcome? To our knowledge, the mechanism of the Arduengo imidazolium salt synthesis has not been discussed in the literature. We propose that alkylation of the DADs **A** by formaldehyde and HCl (or TMSCl) leads to an iminium salt **B** (Scheme 2); analogous alkylations can be induced by chloromethylethers. Loss of HCl from **B** (presumably to **A**, which is a monobasic species)[13] gives an imino-azomethin-ylide 1,5-dipole **C** (only one mesomeric structure shown), which will undergo 1,5-dipolar cyclization (6π electrocyclization) [18–20] to an oxy-imidazoline **D**. Elimination of the oxy group then generates the imidazolium salt **E**. The electrocyclic mechanism proposed here, though not backed up by experimental data, is plausible considering related precedence [18–20] and because it proceeds via a favorable electroneutral intermediate (**C**). Alternative ionic mechanisms require a double alkylation at nitrogen, which leads to an energetically unfavorable dication. While this might be prevented through charge-quenching reversible 1,2-additions of chloride (e.g., to the iminium function in **B**), the resulting mechanistic schemes require additional steps and intermediates which are not backed up by sideproducts.

**Scheme 3:**
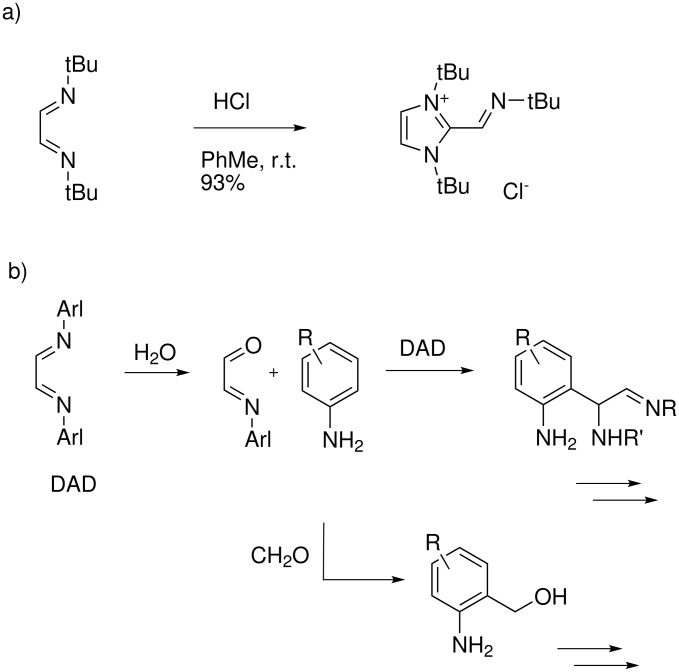
Potential side-reactions in the imidazolium salt synthesis.

The rate-determining step might well be the alkylation of **A** to **B**, and competing processes will lead to side-products. In 1986, tom Dieck and coworkers described that the DAD from *tert*-butylamine reacted with HCl gas in toluene to give an imidazolium salt (Scheme 3a). [21] This is one potential side-reaction, but brown colored side-products can also derive from electrophilic aromatic alkylations of arylamine units by iminium salts from formaldehyde or protonated DADs (as in the bakelite reaction). Such side-reactions will be most prominent with DADs from electron-rich anilines, and with sterically less hindered DADs which are prone to hydrolysis (Scheme 3b). The clean reactions observed under our conditions with the DADs **4–6** are thus probably a consequence of their kinetic stability relative to hydrolysis, in combination with steric hindrance that prevents them from participating as electrophiles in aromatic substitution reactions.

## Conclusion

The useful imidazolium salts IPr·HCl (**1**), IMes·HCl (**2**) and IXy·HCl (**3**) have previously been synthesized by a range of methods giving the products in variable purity and yields. We now present a protocol for the condensation of 1,4-diaryl-diazabutadienes and paraformaldehyde with the following characteristics: a) chlorotrimethylsilane (TMSCl) serves as a cheap and easy to handle source of the chloride counter ion, b) ethyl acetate is the optimal reaction solvent with respect to product purity and precipitation, and c) the reaction requires an appropriate dilution to give high yields. The starting materials and solvents for this synthesis are readily available. The imidazolium salts precipitate from the reaction mixture as pure microcrystalline powders in high yields, considerably surpassing those previously reported in the literature. No product purification is necessary, and the procedure is amenable to large-scale.

## Experimental

A) Synthesis of DAD **4**:[8,10] A solution of glyoxal (72.55 g, 40% in water, 0.50 mol) in MeOH (250 mL) was added with vigorous stirring to a warmed (50°C) solution of 2,6-diisopropylaniline (197 g, purity 90%, 1 mol) and HOAc (1 mL) in MeOH (250 mL). A slightly exothermic reaction commenced and the product started to crystallize after 15 min. The mixture was stirred for 10 h at r.t., after which the resulting suspension was filtered and the solid product washed with MeOH, until the washing phase remained bright yellow. The product was pre-dried by suction over the filter, then dried to constant weight (158.29 g) in high vacuum. The filtrates were collected, evaporated to a volume of 100 mL and set aside for a second crystallization (10.28 g). Total yield: 168.57 g (89.5%) of bright yellow crystals of **4**. The DADs **5** (from 2,6-dimethylaniline, in MeOH, 0.25 mol scale, 2 crops, 84% yield) and **6** (from 2,4,6-trimethylaniline, in iPrOH, 50 mmol scale, 1 crop, 87% yield) were prepared analogously.

B) IPr·HCl (**1**): A 2000 mL round bottom flask containing EtOAc (1200 mL, technical quality, distilled in a rotatory evaporator over K_2_CO_3_) was heated to 70°C in an oil bath. Diazadiene **4** (50.45 g, 134 mmol) and paraformaldehyde (4.06 g, 135 mmol) were added and the walls washed with EtOAc (50 mL). A solution of TMSCl (17.0 mL, 134.5 mmol) in EtOAc (20 mL) was added dropwise over 45 min with vigorous stirring, and the resulting yellow suspension stirred for 2 h at 70°C. After cooling to 10°C (ice-bath) with stirring, the suspension was filtered and the solid washed with EtOAc and tBuOMe. The solid was dried to constant weight in an open dish in a well-ventilated oven at 100°C (1 d), giving 46.04 g (81%) of **1** as colorless microcrystalline powder. The salts IMes·HCl (**2**; 69%) and IXy·HCl (**3**: 89.5%) were prepared analogously, see also Table 1 for conditions.

The reaction products were identified by comparison of ^1^H and ^13^C NMR data to literature values, see Supporting Information File 1 for full experimental data.

## Supporting Information

File 1 General procedures and ^1^H NMR spectra of **1–3**, as obtained from the new synthetic protocol
