# Supplementary material for: Expedient syntheses of the N-heterocyclic carbene precursor imidazolium salts IPr·HCl, IMes·HCl and IXy·HCl
Source: Beilstein J Org Chem. 2007 Aug 28;3:22. doi: 10.1186/1860-5397-3-22 (PMC2151073; doi:10.1186/1860-5397-3-22)

Expedient syntheses of the N-heterocyclic carbene precursor imidazolium salts IPr·HCl, IMes·HCl and IXy·HCl

Lukas Hintermann

Institute of Organic Chemistry
RWTH Aachen University
Landoltweg 1
D-52074 Aachen
Germany

Tel. ++49 (0)241-80-90283
Email: lukas.hintermann@oc.rwth-aachen.de

Additional file 1: supporting information

General information

All reactions were performed in air.

Chemicals

EtOAc: technical grade, distilled in a rotatory evaporater over K_2CO_3. **tBuOMe**: technical grade, distilled. **Paraformaldehyde**: this was a finely powdered material, "for synthesis" grade. **TMSCl**: Merck, "for synthesis". **2,6-Diisopropylaniline**: Fluka or Alfa Aesar, technical grade, 90% specified purity. **2,6-Dimethylaniline**: Fluka, purum 98%. **Glyoxal solution 40% in water**: Alfa Aesar.

Synthesis of 1,4-diazadienes

The synthesis of 1,4-diaryl-1,4-diazadienes is exemplified by the following example:

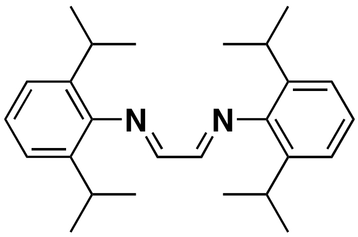

1,4-Bis-(2,6-diisopropylphenyl)-1,4-diaza-butadiene (**4**): A solution of glyoxal (72.55 g, 40% in water, 0.50 mol) in MeOH (250 mL) was added with vigorous stirring to a warmed (50 °C) solution of 2,6-diisopropylaniline (197 g, purity 90%, 1 mol) and HOAc (1 mL) in MeOH (250 mL). A slightly exothermic reaction commenced and the product started to crystallize after 15 min. The mixture was stirred for 10 h at r.t., after which the resulting suspension was filtered and the solid product washed with MeOH, until the washing phase remained bright yellow. The product was pre-dried by suction over the filter, then dried to constant weight (158.29 g) in high vacuum. The filtrates were collected, evaporated to a volume of 100 mL and set aside for a second crystallization (10.28 g). Total yield: 168.57 g (89.5%) of bright yellow crystals of **4**.

The DADs **5** (from 2,6-dimethylaniline, in MeOH, 0.25 mol scale, 2 crops, 84% yield) and **6** (from 2,4,6-trimethylaniline, in iPrOH, 50 mmol scale, 1 crop, 87% yield) were prepared analogously.

The products were identified by comparison of spectral data to the literature:

4: a) A. J. Arduengo, R. Krafczyk, R. Schmutzler, H. A. Craig, J. R. Goerlich, W. J. Marshall, M. Unverzagt, *Tetrahedron* **1999**, *55*, 14523-14534. b) L. Jafarpour, E. D. Stevens, S. P. Nolan, *J. Organomet. Chem.* **2000**, *606*, 49–54.

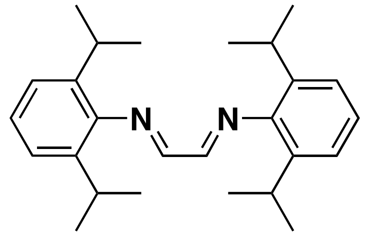

5: T. M. Trnka, J. P. Morgan, M. Sanford, T. E. Wilhelm, M. Scholl, T.-L. Choi, S. Ding, M. W. Day, R. H. Grubbs, *J. Am. Chem. Soc.* **2003**, *125*, 2546–2558.

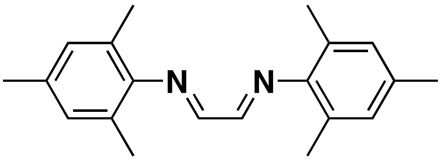

6: H. tom Dieck, M. Svoboda, T. Greiser, *Z. Naturforsch. B* **1981**, *36*, 823–832.

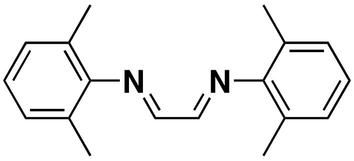

Synthesis of imidazolium salts

The synthesis of 1,3-Diaryl-imidazolium chlorides is exemplified by the following example:

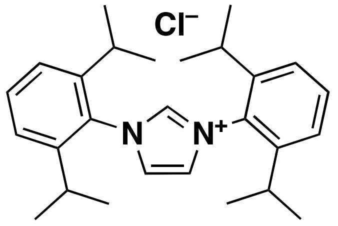

1,3-Bis-(2,6-diisopropylphenyl)-imidazolium chloride (IPr·HCl, **1**): A 2000 mL round bottom flask containing EtOAc (1200 mL, technical grade, distilled in a rotatory evaporator over K_2CO_3) was heated to 70 °C in an oil bath. Diazadiene **4** (50.45 g, 134 mmol) and paraformaldehyde (4.06 g, 135 mmol) were added and the walls washed with EtOAc (50 mL). A solution of TMSCl (17.0 mL, 134.5 mmol) in EtOAc (20 mL) was added dropwise over 45 min with vigorous stirring, and the resulting yellow suspension stirred for 2 h at 70 °C. After cooling to 10 °C (ice-bath) with stirring, the suspension was filtered and the solid washed with EtOAc and tBuOMe. The solid was dried to constant weight in an open dish in a well-ventilated oven at 100 °C (1 d), giving 46.04 g (81%) of **1** as colorless microcrystalline powder.
A dried (120 °C) sample of **1** (139.9 mg) was stored open in air for a day; it absorbed 3.1 mg of water, corresponding to 0.5 molar equivalents. The water can be removed by drying at 100 °C in an oven.

The salts IMes·HCl (**2**; 69%; yellow aspect) and IXy·HCl (**3**: 89.5%) were prepared analogously, see Table 1 of the main text for conditions.

The products were characterized spectroscopically and identified by comparison to literature data:

1: A. J. Arduengo, R. Krafczyk, R. Schmutzler, H. A. Craig, J. R. Goerlich, W. J. Marshall, M. Unverzagt, *Tetrahedron* **1999**, *55*, 14523–14534.

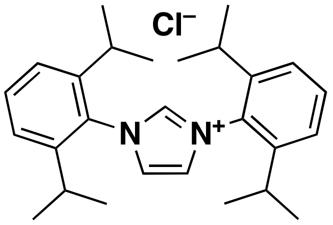

2: A. J. Arduengo, R. Krafczyk, R. Schmutzler, H. A. Craig, J. R. Goerlich, W. J. Marshall, M. Unverzagt, *Tetrahedron* **1999**, *55*, 14523–14534.

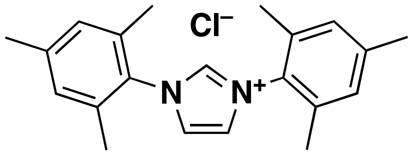

3: L. Delaude, M. Szypa, A. Demonceau, A. F. Noels, *Adv. Synth. Catal.* **2002**, *344*, 749–756.

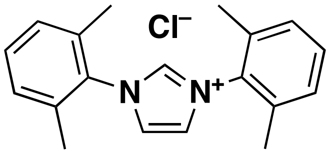

^{1}H NMR Spectra of imidazolium salts as obtained by the new method

(see following pages)

*^{1}H NMR spectrum of IPr·HCl (**1**) in DMSO-d6, 300 MHz:*

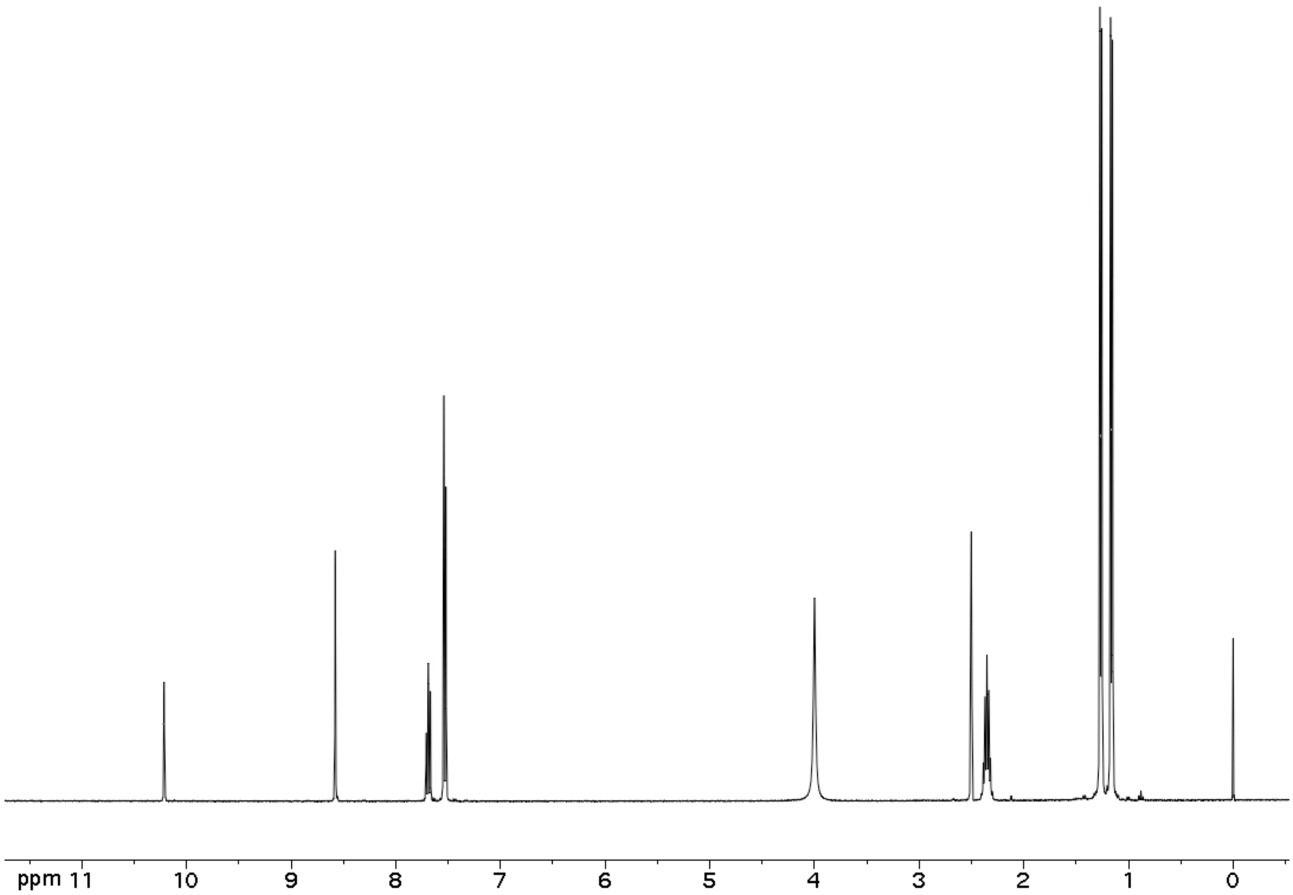

*^{1}H NMR spectrum of IMes·HCl (**2**) in $CDCl_3$, 400 MHz:*

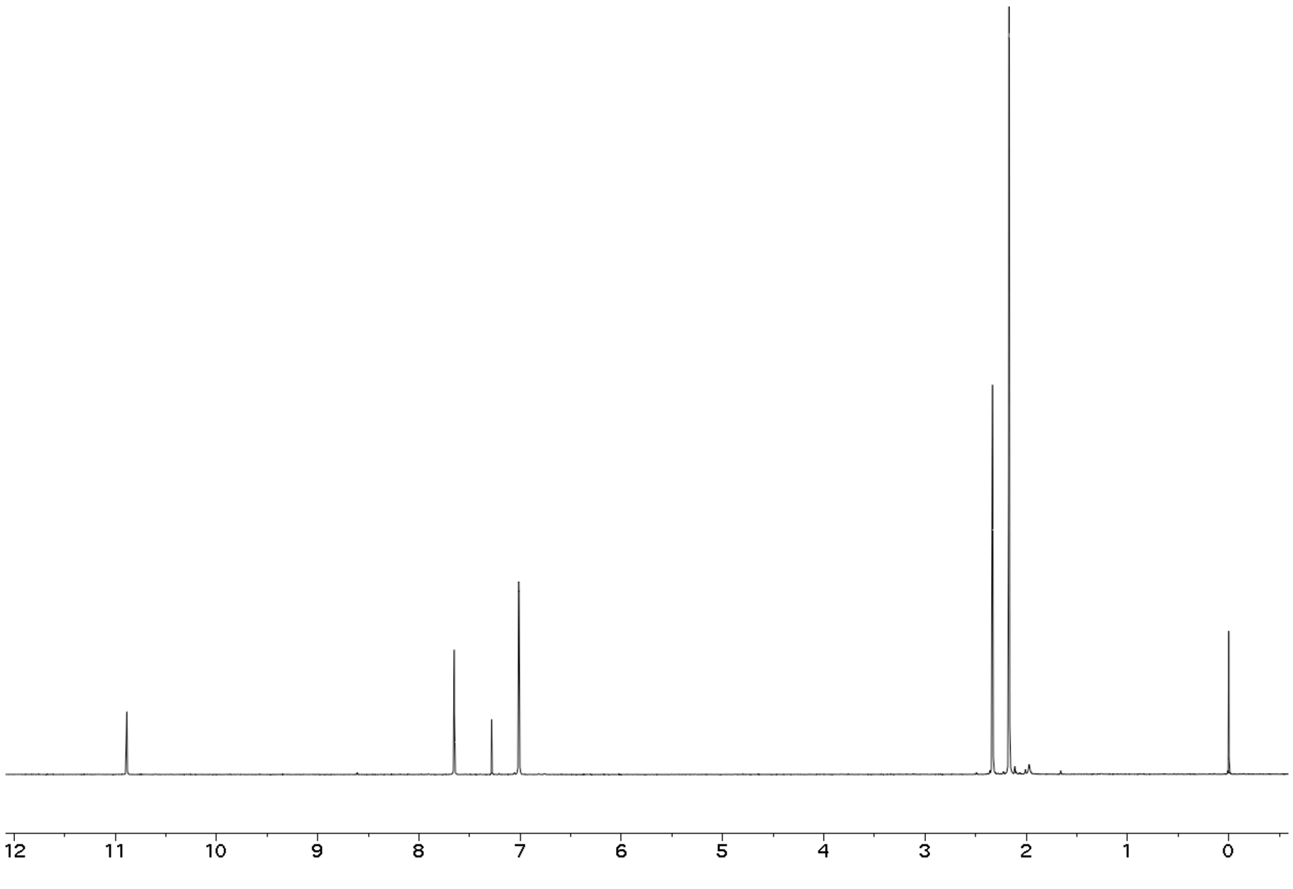

*^{1}H NMR spectrum of IXy·HCl (**3**) in DMSO-d6, 300 MHz:*

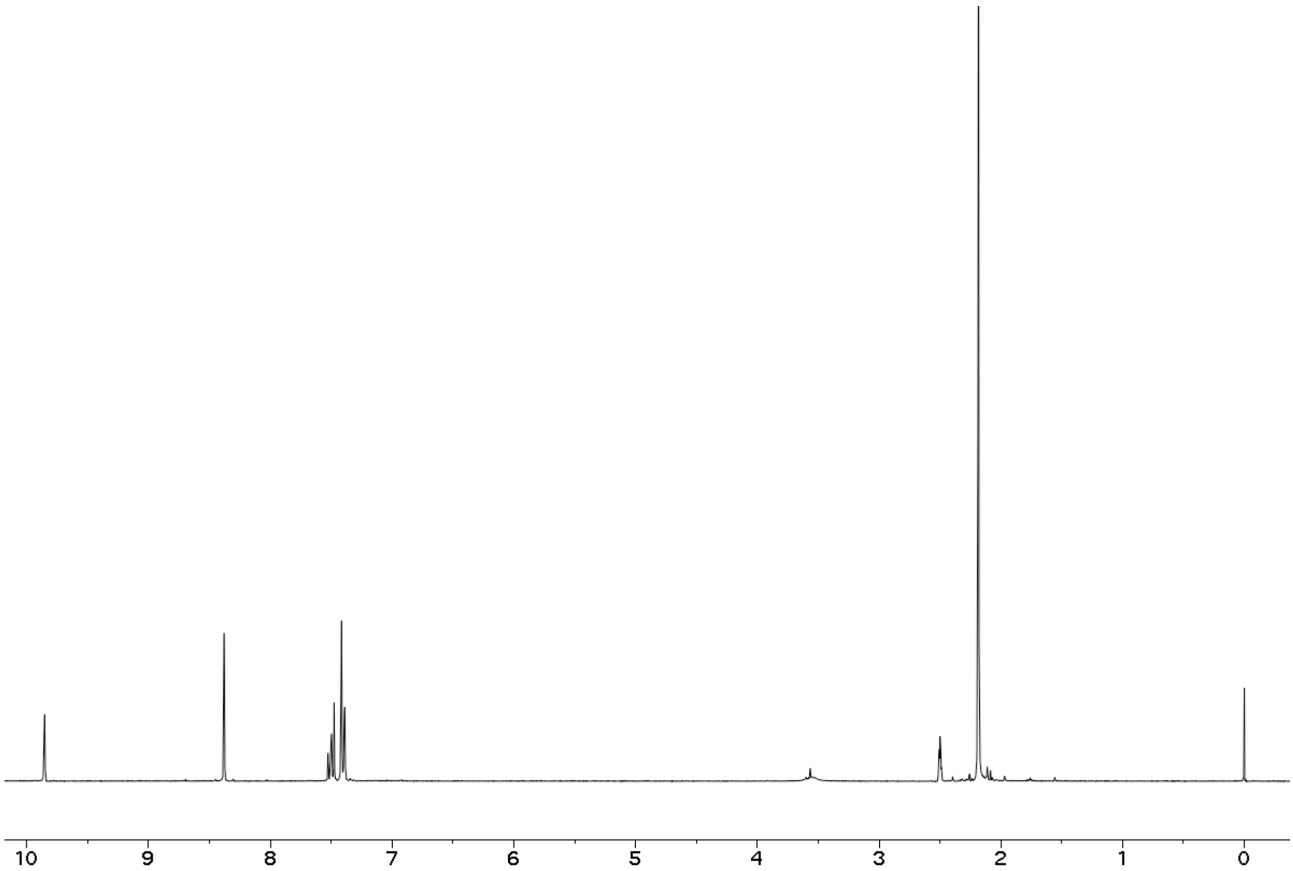

*^{1}H NMR spectrum of IXy·HCl (**3**) in $CDCl_3$, 400 MHz:*

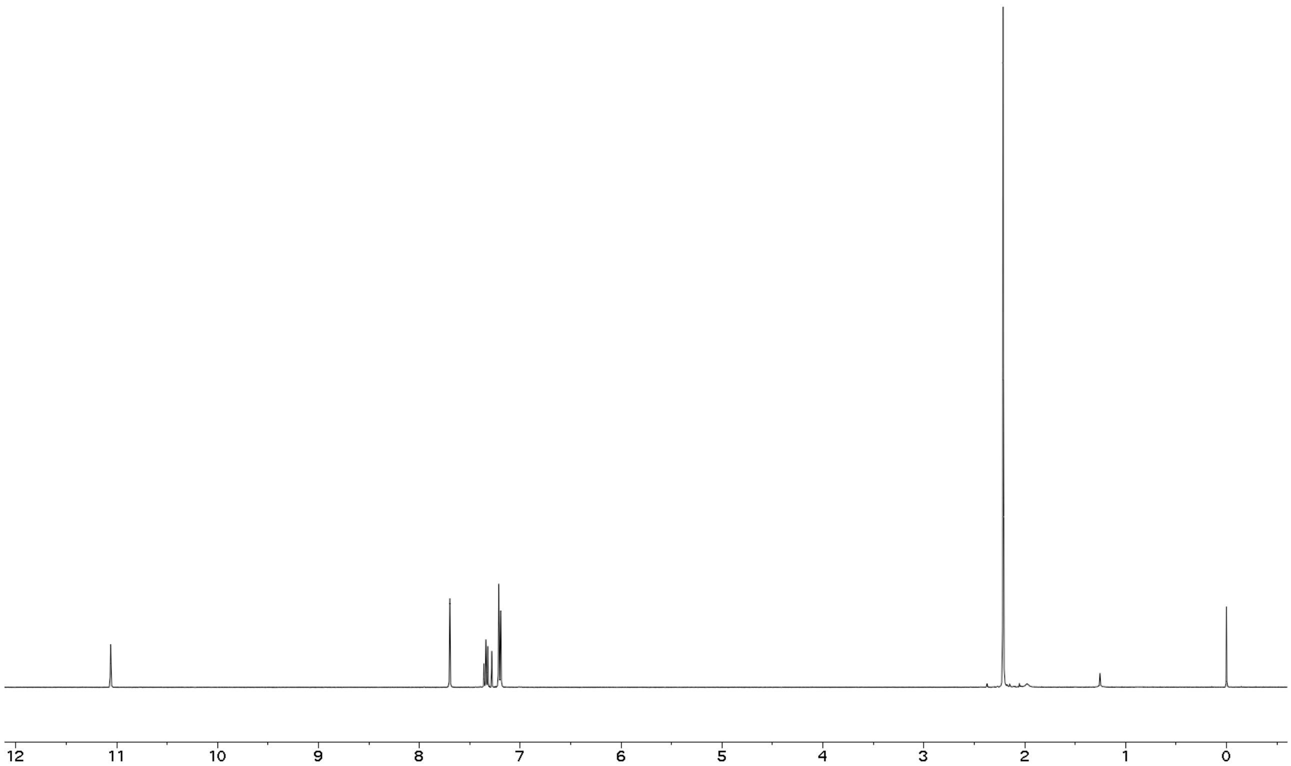

Supplement: File 1 — General procedures and 1H NMR spectra of 1–3, as obtained from the new synthetic protocol [file Beilstein_J_Org_Chem-03-22-s001.pdf]
